# Supplementary material for: Effects of pericapsular nerve group block versus local anesthetic infiltration for postoperative analgesia in total hip arthroplasty: A protocol for systematic review and meta-analysis
Source: PLoS One. 2025 Mar 10;20(3):e0319102. doi: 10.1371/journal.pone.0319102 (PMC11893113; doi:10.1371/journal.pone.0319102)
Supplement: S2 File — (DOCX) [file pone.0319102.s002.docx]

**Search strategies**

**PubMed**

#1 pericapsular nerve group block [Title/Abstract]

#2 PENG block [Title/Abstract]

#3 #1 OR #2

#4 local infiltration analgesia [Title/Abstract]

#5 local anesthetic infiltration [Title/Abstract]

#6 local infiltration anesthesia [Title/Abstract]

#7 #4 OR #5 OR #6

#8 randomized controlled trial [Title/Abstract]

#9 randomized controlled study [Title/Abstract]

#10 controlled clinical trial [Title/Abstract]

#11 clinical study [Title/Abstract]

#12 #8 OR #9 OR #10 OR #11

#13 #3 AND #7 AND #12

**EMBASE**

#1 ‘pericapsular nerve group block’:ti,ab,kw OR ‘PENG block’:ti,ab,kw

#2 ‘local infiltration analgesia’:ti,ab,kw OR ‘local anesthetic infiltration’:ti,ab,kw OR ‘local infiltration anesthesia’:ti,ab,kw

#3 ‘randomized controlled trial’/exp OR ‘randomized controlled trial’:ti,ab,kw OR

‘randomized controlled study’:ti,ab,kw OR ‘controlled clinical trial’:ti,ab,kw OR ‘clinical study’:ti,ab,kw

#4 #1 AND #2 AND #3

**Cochrane Library**

#1 (pericapsular nerve group block):ti,ab,kw

#2 (PENG block):ti,ab,kw

#3 #1 OR#2

#4 (local infiltration analgesia):ti,ab,kw

#5 (local anesthetic infiltration):ti,ab,kw

#6 (local infiltration anesthesia):ti,ab,kw

#7 #4 OR #5 OR #6

#8 (randomized controlled trial):ti,ab,kw

#9 (randomized controlled study):ti,ab,kw

#10 (controlled clinical trial):ti,ab,kw

#11 (clinical study):ti,ab,kw

#12 #8 OR #9 OR #10 OR #11

#13 #3 AND #7 AND #12

**Web of Science**

#1 pericapsular nerve group block (Topic) OR PENG block (Topic)

#2 local infiltration analgesia (Topic) OR local anesthetic infiltration (Topic) OR local infiltration anesthesia (Topic)

#3 randomized controlled trial (Topic) OR randomized controlled study (Topic) OR

controlled clinical trial (Topic) OR clinical study (Topic)

#4 #1 AND #2 AND #3
