# Supplementary material for: Effects of pericapsular nerve group block versus local anesthetic infiltration for postoperative analgesia in total hip arthroplasty: A protocol for systematic review and meta-analysis
Source: PLoS One. 2025 Mar 10;20(3):e0319102. doi: 10.1371/journal.pone.0319102 (PMC11893113; doi:10.1371/journal.pone.0319102)
Supplement: Attachment — Submitted filename: Response to Reviewers.docx [file pone.0319102.s004.docx]

**Responses to Reviewers/Editor Letter for revision of PONE-D-24-45094**

Dear Dr. Robert Jeenchen Chen and Reviewers (Dr. Syed Azfar and reviewer #2),

Thank you very much for giving us the opportunity to revise our manuscript (PONE-D-24-45094). We appreciate the helpful feedback from you and the reviewers. After carefully reading the comments, we have revised the manuscript point-by-point. Herewith we resubmit a revised manuscript for your assessment. Important changes are highlighted, and detailed responses to each comment are included below. We believe that these revisions have substantially improved the manuscript, and we thank you and the reviewers for their thoughtful comments, which are really helpful for the improvement. All authors of the manuscript have read the revised manuscript prior to re-submission and agree with its contents.

We greatly appreciate your consideration of our manuscript and look forward to hearing from you and reviewers.

**Journal Requirements:**

When submitting your revision, we need you to address these additional requirements.

1. Please ensure that your manuscript meets PLOS ONE's style requirements, including those for file naming. The PLOS ONE style templates can be found at

https://journals.plos.org/plosone/s/file?id=wjVg/PLOSOne_formatting_sample_main_body.pdf and

https://journals.plos.org/plosone/s/file?id=ba62/PLOSOne_formatting_sample_title_authors_affiliations.pdf

Response: Thank you! We have checked our manuscript and ensure it meets PLOS ONE's style requirements.

2. Thank you for stating the following financial disclosure:

"This work was supported by the Guangdong Basic and Applied Basic Research Foundation (grant No. 2024A1515012880, YW)."

Please state what role the funders took in the study. If the funders had no role, please state: ""The funders had no role in study design, data collection and analysis, decision to publish, or preparation of the manuscript.""

If this statement is not correct you must amend it as needed.

Please include this amended Role of Funder statement in your cover letter; we will change the online submission form on your behalf.

Response: Thank you! We have stated what role the funders took in the study (page 11, line 12-14).

**Reviewers' comments:**

**Reviewer #1:** 1) The manuscript outlines a rationale by highlighting the conflicting results in existing studies regarding the efficacy of pericapsular nerve group (PENG) block versus local anesthetic infiltration for postoperative pain control in total hip arthroplasty. The study aims to perform a systematic review and meta-analysis to synthesize available evidence, addressing a significant gap in the literature and contributing to the optimization of pain management strategies in orthopedic surgery. A similar meta analysis comparing placebo Vs LIA vs PENG was done previously in 2016.

(Jiménez-Almonte, et. al, Is Local Infiltration Analgesia Superior to Peripheral Nerve Blockade for Pain Management After THA: A Network Meta-analysis. Clinical Orthopaedics and Related Research 474(2):p 495-516, February 2016. | DOI: 10.1007/s11999-015-4619-9)

Response: Thank you very much for this encouraging comments. In this revision, we have described and cited this study (PMID: 26573322) in this revision (page 4, line 20-22).

2) The protocol outlines appropriate databases and search strategies, including clear inclusion and exclusion criteria. The study design aligns with PRISMA-P guidelines, ensuring methodological rigor. However, potential limitations, such as language restrictions and variations in analgesic types and outcome definitions, may impact the reliability of results. These aspects are acknowledged but could benefit from a more comprehensive strategy for mitigating bias.

Response: We thank the reviewer for this good comment. We completely agree with the reviewer that these potential limitations, including language restrictions and variations in analgesic types and outcome definitions will impact the reliability of combined results. Subgroup analysis will further be performed to explore the source of heterogeneity, and sensitivity analysis will be performed to test the reliability of combined results. We have described this and provided more information for mitigating bias (page 8, line 4-18).

3) The methodology includes detailed criteria for study selection, data extraction, and statistical analysis. The protocol specifies the use of established tools (e.g., RevMan 5.4) for meta-analysis, risk assessment methods, and criteria for handling heterogeneity. The detailed approach enhances reproducibility.

Response: Thank you very much for this encouraging comments.

4) The manuscript states that no datasets were generated during the protocol’s development and that relevant data will be available upon study completion. However, it does not specify the exact repository or access details for data sharing, which is required for transparency and compliance with data availability policies.

Response: Thank you for this nice comment. For this protocol, no datasets have been generated and/or analyzed. When the related meta-analysis of this protocol is finished, we will submit the results and data to one peer-reviewed journal and published it publicly. We have made a Data Availability Statement in this revision (page 11, line 19-24).

5) The protocol is clearly written, with standard scientific English and minimal typographical errors. The structure follows accepted guidelines for study protocols, enhancing readability and comprehension.

Response: Thank you very much.

6) The manuscript provides a solid foundation for a systematic review and meta-analysis aimed at resolving discrepancies in postoperative analgesia strategies for total hip arthroplasty.

Response: Thank you very much for this nice comment.

However, there are a few areas for improvement:

a) Data Availability: Specify the repository and data access policies to ensure transparency.

Response: Thank you. We have specified the repository and data access policies to ensure transparency (page 11, line 19-24).

b) Mitigation of Bias: Address limitations related to language restrictions and heterogeneity more comprehensively, potentially by planning subgroup analyses or sensitivity checks.

Response: Thank you very much for this helpful suggestion. We have described how to address limitations related to language restrictions and heterogeneity more comprehensively by planning subgroup analyses and sensitivity analysis (page 8, line 4-18).

c) Exploratory Aspects: Consider discussing how exploratory analyses will be handled, particularly if unexpected patterns or variations in outcomes are found.

Response: Thank you very much. We have discussed how exploratory analyses will be handled as “Exploratory Data Analysis might be performed when encountering unexpected patterns or variations in outcomes. For example, different types of analgesics might be used across included studies, and we will unify the analgesic consumption to morphine equivalent consumption according to the methods described in one previous paper (PMID: 18574361). For data that presented using the median and range, we will convert them to the mean and standard deviation (PMID: 15840177). If there were two interventions groups in one included study, we will combine them into one intervention group (Cochrane Handbook for Systematic Reviews of Interventions Version 5.1.0.). If pain scores were not reported clearly at rest or on movement, we will contact the authors.” (page 10, line 18-26).

Overall, the study protocol appears fair, with potential to contribute good insights into pain management techniques for orthopedic surgery.

Response: Thank you very much for your valuable comments, which have substantially improved our manuscript.

**Reviewer #2:** 1. This research has a certain degree of innovation and has certain reference value for clinical practice. However, the manuscript is very brief and there are few references. It is recommended to further enrich the content. For example, subgroup analysis, sensitivity analysis, publication bias analysis, and the method of GRADE assessment of quality grades should be introduced in detail in separate paragraphs.

Response: Thank you very much. We have added detailed information for subgroup analysis, sensitivity analysis, publication bias analysis, and the GRADE methods (page 8, line 4-28; page 9, line 1-14). Moreover, we have described how to handle unexpected patterns or variations in outcomes (page 10, line 18-26) and added more references as recommended (page 4, line 7, 9, 20 and 22).

2. Please describe in detail what you did when you encountered a study with incomplete information during the study selection process, such as whether you tried to determine whether it met the inclusion criteria by obtaining more information, such as contacting the authors.

Response: Thank you very much for this good question. When we encountered a study with incomplete information during the study selection process, we will contact the authors for additional information to determine whether the study meet the inclusion criteria. We have described this (page 7, line 3-5). Furthermore, we have described how to handle unexpected patterns or variations in outcomes (page 10, line 18-26)

3. Please provide the retrieval formula construction process for each database, not just an example of the PubMed retrieval plan, to demonstrate the comprehensiveness and scientific nature of the retrieval.

Response: Thank you for this helpful suggestion. We have included the full search strategy for all databases in a supplementary file (S2_File). Accordingly, this supplementary file has be cited in the main text (page 6, line 15).
