# Supplementary material for: Effects of pericapsular nerve group block versus local anesthetic infiltration for postoperative analgesia in total hip arthroplasty: A protocol for systematic review and meta-analysis
Source: PLoS One. 2025 Mar 10;20(3):e0319102. doi: 10.1371/journal.pone.0319102 (PMC11893113; doi:10.1371/journal.pone.0319102)
Supplement: Attachment — Submitted filename: Response_to_Reviewers_auresp_2.docx [file pone.0319102.s005.docx]

**Responses to Reviewers/Editor Letter for revision of PONE-D-24-45094R1**

Dear Dr. Robert Jeenchen Chen and Reviewers,

Thank you very much for giving us the opportunity to revise our manuscript (PONE-D-24-45094R1). We appreciate the helpful feedback from you and the reviewers. After carefully reading the comments, we have revised the manuscript point-by-point. Herewith we resubmit a revised manuscript for your assessment. Important changes are highlighted, and detailed responses to each comment are included below. We believe that these revisions have substantially improved the manuscript, and we thank you and the reviewers for their thoughtful comments, which are really helpful for the improvement. All authors of the manuscript have read the revised manuscript prior to re-submission and agree with its contents.

We greatly appreciate your consideration of our manuscript and look forward to hearing from you and reviewers.

**Journal Requirements:**

Please review your reference list to ensure that it is complete and correct. If you have cited papers that have been retracted, please include the rationale for doing so in the manuscript text, or remove these references and replace them with relevant current references. Any changes to the reference list should be mentioned in the rebuttal letter that accompanies your revised manuscript. If you need to cite a retracted article, indicate the article’s retracted status in the References list and also include a citation and full reference for the retraction notice.
Response: Thank you! We have checked the reference list to ensure that it is complete and correct.

**Reviewers' comments:**

**Reviewer #1:** Please accept revised version of submission. As most of the points are well taken and revised as per suggestions.

Response: Thank you very much for your valuable comments, which have substantially improved our manuscript.

**Reviewer #2:** Appreciate the efforts of the authors. The manuscript has been well revised, but there are still some issues that need to be addressed.

1. The description of the exclusion criteria could be more detailed. For example, in addition to the studies that do not meet the inclusion criteria, whether certain specific types of studies are also excluded (such as those with an extremely small sample size or extremely low quality), and the specific reasons for the exclusion. Consider whether it is necessary to set some additional exclusion criteria, such as studies that have been repeatedly published, studies for which the full text cannot be obtained, etc., in order to further optimize the quality of the included studies.
Response: We thank the reviewer for these nice suggestions. We have added this information in the manuscript as “Studies do not meet above-mentioned criteria will be included, which include the following study types: retrospective studies, systematic review and meta-analysis, narrative reviews, conference abstracts, case reports, comments, letters, perspectives, insights, correspondences, and editorials. Moreover, in order to further optimize the quality of included studies, studies with an extremely small sample size or extremely low quality or unavailable full-text, and repeatedly published studies will be excluded.” (page 6, line 26-28; page 7, line 1-3).

2. If publication bias is found to exist, what measures will be taken to adjust or explain it? For example, conducting a trim-and-fill analysis or exploring the extent to which publication bias affects the research results, so as to enhance the credibility of the research results.
Response: Thank you very much for this good question. We have described how to handle publication bias as “A skewed funnel indicates publication bias exists, and a ‘trim and fill’ method will be further used to correct the funnel plot asymmetry by 1) removing the smaller studies causing asymmetry, 2) using the trimmed funnel plot to evaluate the true funnel center, and 3) replacing the omitted studies around the funnel center.” (page 9, line 1-5).

3. Discussion Section.
In addition to the limitations already mentioned, other factors that may affect the research results can also be considered, such as regional differences in the research (the medical level and patient characteristics may vary in different regions), differences in follow-up time (the assessment of long-term analgesic effects and complications may be insufficient), etc., and discuss how to make improvements in subsequent studies.
Response: Thank you very much for pointing out this issue. We completely agree with the reviewer that other factors that may affect the research results, such as regional differences and differences in follow-up time, we have added this information. Subgroup group analysis and sensitivity analysis will be further conducted to identify their influence on the pooled results. For subsequent studies, it’s better to include patients with similar medical levels, characteristics, and follow-up time. We have discussed this issue in the revision (page 10, line 9-10, line 13-17).

You mentioned in the text that the usage amount of analgesics will be uniformly converted into the morphine equivalent usage amount. Please elaborate on how you will carry out this conversion.

Response: Thank you very much. We have provided the conversion formula for commonly used analgesics and cited the relevant references (page 10, line 24-26).

Finally, thank you again for your great help in improving our manuscript.
