# Supplementary material for: Effects of pericapsular nerve group block versus local anesthetic infiltration for postoperative analgesia in total hip arthroplasty: A protocol for systematic review and meta-analysis
Source: PLoS One. 2025 Mar 10;20(3):e0319102. doi: 10.1371/journal.pone.0319102 (PMC11893113; doi:10.1371/journal.pone.0319102)
Supplement: Attachment — Submitted filename: Response_to_Reviewers_auresp_3.docx [file pone.0319102.s006.docx]

**Responses to Reviewers/Editor Letter for revision of PONE-D-24-45094R2**

Dear Dr. Robert Jeenchen Chen and Reviewers,

Thank you very much for giving us the opportunity to revise our manuscript (PONE-D-24-45094R2). We appreciate the helpful feedback from you and the reviewers. After carefully reading the comments, we have revised the manuscript point-by-point. Herewith we resubmit a revised manuscript for your assessment. Important changes are highlighted, and detailed responses to each comment are included below. We believe that these revisions have substantially improved the manuscript, and we thank you and the reviewers for their thoughtful comments, which are really helpful for the improvement. All authors of the manuscript have read the revised manuscript prior to re-submission and agree with its contents.

We greatly appreciate your consideration of our manuscript and look forward to hearing from you and reviewers.

**Journal Requirements:**

Please review your reference list to ensure that it is complete and correct. If you have cited papers that have been retracted, please include the rationale for doing so in the manuscript text, or remove these references and replace them with relevant current references. Any changes to the reference list should be mentioned in the rebuttal letter that accompanies your revised manuscript. If you need to cite a retracted article, indicate the article’s retracted status in the References list and also include a citation and full reference for the retraction notice.
Response: Thank you! We have checked the reference list to ensure that it is complete and correct.

**Reviewers' comments:**

**Reviewer #3:** Thank you very much for allowing me to review your manuscript. I appreciate your effort in performing this study and submitting it to plos one. I would like to point out a few things that should be corrected:
1) I assume the author should rewrite the inclusion part in a better format. In addition, you add a sentence that should change it. You wrote: “Studies do not meet above-mentioned criteria will be included”. I think you wanted to write excluded instead of included.

Response: Thank you very much for your valuable comments. During last revision stage, we have updated the inclusion and exclusion criteria as “Inclusion criteria: 1) Study type: RCTs; 2) Participants: patients underwent total hip arthroplasty; 3) Interventions: pericapsular nerve group block; 4) Control: local anesthetic infiltration; 5) Primary outcomes: the time to first analgesics requirement (analgesia duration); Secondary outcomes: postoperative analgesics consumption over 24 hours, visual analog scale (VAS) scores at rest and movement, and the incidence of adverse effects. Studies do not meet above-mentioned criteria will be excluded, which include the following study types: retrospective studies, systematic review and meta-analysis, narrative reviews, conference abstracts, case reports, comments, letters, perspectives, insights, correspondences, and editorials. Moreover, in order to further optimize the quality of included studies, studies with an extremely small sample size or extremely low quality or unavailable full-text, and repeatedly published studies will be excluded.”. (page 6, line 17-28)

2) The PRISMA chart you included is a template and I cannot see the number of studies you included or excluded in your PRISMA chart.

Response: Thank you very much for this nice question. This meta-analysis has not yet started, therefore, we did not include the number of studies in the PRISMA chart. It is normal for a protocol and is consistent with the journal guidelines of Plos One. Thanks for your understanding.

3) Your discussion part is too short. Please extend it.

Response: Thank you very much. During the revision, we have extended the discussion part as “Although increasing numbers of studies have compared the effects of PENG block with local anesthetic infiltration on postoperative pain for total hip arthroplasty, no meta-analysis has yet provided synthesized evidence. This study aimed to provide a protocol to determine whether PENG block is superior to local anesthetic infiltration in controlling postoperative pain for total hip arthroplasty. There might be several limitations when conducting this meta-analysis. First, substantial heterogeneity between included studies resulting from the type of local anesthetics, the definition of primary outcomes, the combination drugs, regional differences regarding medical levels and patients’ characteristics, differences in follow-up time, etc., will influence the reliability of pooled results. Second, the number of RCTs that directly compared the effects of PENG block to local anesthetic infiltration might be small. Finally, publication bias might exist because the English language restriction. To minimize the influence of heterogeneity, subgroup analysis will be used to explore their source, and sensitivity analysis will be performed to assess the reliability of pooled data. For subsequent studies, it’s better to include patients with similar baselines, including medical levels, characteristics, and follow-up time, etc. Furthermore, the GRADE approach will be conducted to rank the evidence quality for major outcomes. This protocol has been registered in the PROSPERO and was generated according to the PRISMA-P guidelines.

Exploratory Data Analysis might be performed when encountering unexpected patterns or variations in outcomes. For example, different types of analgesics might be used across included studies, and we will unify the analgesic consumption to morphine equivalent consumption according to the methods described in previous papers (e.g., morphine 1 mg, iv. = tramadol 10 mg, iv. = fentanyl 10 mcg, iv. = sufentanil 1 mcg, iv = pethidine 10 mg, iv = oxycodone 1.5 mg, oral). For data that presented using the median and range, we will convert them to the mean and standard deviation. If there were two interventions groups in one included study, we will combine them into one intervention group (Cochrane Handbook for Systematic Reviews of Interventions Version 5.1.0.). If pain scores were not reported clearly at rest or on movement, we will contact the authors.”. (page 10-11)

**Reviewer #4:** The efforts of the authors is praiseworthy.

Response: Thank you very much for your encouraging comment.

Kindly shorten the introduction section of abstract. It is lengthy.

Response: Thank you very much for this helpful suggestion. We have shortened the introduction section of abstract as “This protocol for a systematic review and meta-analysis aims to provide synthesized evidence to determine whether pericapsular nerve group (PENG) block is superior to local anesthetic infiltration in controlling postoperative pain in total hip arthroplasty.”. (page 2, line 2-5)

Finally, thank you again for your great help in improving our manuscript.
